# Exposure to amitraz, fipronil and permethrin affects cell viability and ABC transporter gene expression in an *Ixodes ricinus* cell line

**DOI:** 10.1186/s13071-018-3020-4

**Published:** 2018-07-31

**Authors:** Carlo Mangia, Alice Vismarra, Marco Genchi, Sara Epis, Claudio Bandi, Giulio Grandi, Lesley Bell-Sakyi, Domenico Otranto, Benedetta Passeri, Laura Kramer

**Affiliations:** 1 Department of Veterinary Sciences, University of Parma, 43126 Parma, Italy; 2 Department of Veterinary Sciences and Public Health, University of Milan, 20133 Milan, Italy; 3 Department of Biosciences, University of Milan, 20133 Milan, Italy; 4 Department of Biomedical Sciences and Veterinary Public Health (BVF), Swedish University of Agricultural Sciences (SLU), SE-757 56 Uppsala, Sweden; 5 Department of Infection Biology, Institute of Infection and Global Health, University of Liverpool, Liverpool, L3 5RF UK; 6 Department of Veterinary Medicine, University of Bari, 70010 Valenzano, Bari Italy; 7 Pediatric Clinical Research Center Romeo and Enrica Invernizzi, Ospedale “Luigi Sacco”, 20157 Milan, Italy

**Keywords:** Acaricide, Amitraz, Fipronil, Permethrin, *Ixodes ricinus*, *In vitro*, Tick cell line, Trypan blue, MTT assay

## Abstract

**Background:**

Over-expression of ATP-binding cassette (ABC) transporter proteins has been implicated in resistance of ticks to acaricides. Tick cell lines are useful for investigating resistance mechanisms, as development of an *in vitro* model for the study of acaricide resistance would contribute to improving knowledge of the molecular basis behind drug processing and exclusion in ticks. In the present study, cultures of the *Ixodes ricinus*-derived cell line IRE/CTVM19 were treated with the acaricides amitraz, permethrin or fipronil to determine modulation of ABC transporter gene expression. Cells were treated with different drug concentrations (25, 50, 100, 150 μM) and incubated for ten days. Cell morphology, viability, metabolic activity and relative expression of ABC (B1, B6, B8 and B10) genes were determined at day 10 post-treatment.

**Results:**

Cell morphology determined by light microscopy was altered following treatment with all drugs, but only at high concentrations, while total cell numbers decreased with increasing drug dose. Cell viability determined by trypan blue exclusion was not significantly different from untreated controls (*P* > 0.1) following treatment with amitraz and permethrin, but high concentrations of fipronil caused decrease (up to 37%, *P* < 0.01) in viability. At all drug concentrations, fipronil and permethrin induced dose-dependent reduction in cell metabolic activity measured by MTT assay (*P* < 0.01). Quantitative RT-PCR showed that the drugs significantly affected expression of ABC genes. In particular, fipronil treatment downregulated ABCB1 (*P* < 0.001) and upregulated ABCB6, ABCB8 and ABCB10 (*P* < 0.01); amitraz treatment down regulated ABCB1 (significant difference between 25 and 150 μM, *P* < 0.001) and upregulated ABCB8 and ABCB10 at lower concentrations (25 and 50 μM, *P* < 0.05); and permethrin upregulated ABCB6, ABCB8 and ABCB10 only at 150 μM (*P* < 0.01).

**Conclusions:**

The adverse effects on cell viability and metabolic activity, and changes in expression of different ABC transporter genes, detected in IRE/CTVM19 cells following treatment with amitraz, permethrin and fipronil, support the proposed application of tick cell lines as *in vitro* models for the study of resistance to these acaricides in ticks.

## Background

Ticks are among the most important vectors of pathogens affecting livestock, companion animals and humans worldwide [1]. Tick-borne pathogens (TBPs) of livestock cause morbidity and mortality with consequent reduction of milk and meat production, resulting in reported worldwide losses of over 14 billion USD per year in cattle [2]. Infection of companion animals and livestock with TBPs can cause severe disease and several are zoonotic; prevention is essential for safeguarding public health and protecting the human-animal bond [3, 4], making TBPs one of the most important issues in the “One World, One Health” concept [5].

Tick control depends mainly on the use of chemical acaricides on animals and/or in the environment. However, there are increasing reports of lack of efficacy of/resistance to several commonly-used acaricides, including permethrin, fipronil and amitraz [6, 7]. Even when reduced sensitivity to acaricides is observed in the field, loss of acaricide activity should be confirmed through *ex vivo* testing on different tick developmental stages (e.g. adult immersion assay, larval packet test, etc.) [8]. Tick cell lines have been recently investigated for their potential in studying acaricide resistance and, specifically, to elucidate the molecular mechanism(s) underlying the lack of efficacy of acaricides [9–11].

The ATP-binding cassette (ABC) transporters (ABCTs) are membrane proteins that participate in the transport of drugs and metabolites across cell membranes, often against their concentration gradient [12]. They have been implicated in the development of resistance to chemotherapeutics in cancer patients [13] and, more recently, in resistance of mosquitoes to insecticides [14, 15] and of different helminths to anthelmintic drugs [16, 17]. Studies carried out in *Rhipicephalus microplus* ticks have shown that overexpression of ABCT genes is associated with resistance to the drug ivermectin [18], and upregulation of several transporter genes follows exposure to ivermectin [19].

The possible involvement of ABCTs in lack of efficacy of ivermectin against the brown dog tick *Rhipicephalus sanguineus* (*sensu lato*) was also demonstrated when an ABCT inhibitor was used in *ex vivo* assays [20]. More recently, treatment of an *Ixodes ricinus*-derived cell line with ivermectin did not result in significant modulation of gene expression for several ABCTs [9].

Amitraz, fipronil and permethrin are among the acaricides most commonly used against tick infestation [21]. They all target the arthropod nervous system, specifically an α-adrenergic receptor agonist (amitraz), a receptor antagonist of γ-aminobutyric acid (GABA)-gated channels (fipronil), or an inhibitor of gated sodium cellular channels (permethrin). In order to further elucidate the role of ABCTs in the response of tick cells to a variety of acaricides, the present study evaluated cell viability and metabolic activity and ABC gene expression (ABCB1, ABCB6, ABCB8, ABCB10) in an *I. ricinus* cell line following treatment with amitraz, fipronil or permethrin.

## Methods

### Cell line maintenance and treatment

The IRE/CTVM19 cell line is derived from the embryonic stage of *I. ricinus* [22]*.* Cells were seeded in flat-sided culture tubes (Nunc™, Thermo Scientific, Milan, Italy) and maintained at 28 °C in Leibovitz’s L-15 medium (Life Technologies, Milan, Italy) supplemented with 10% tryptose phosphate broth, 20% fetal bovine serum, 2mM L glutamine, penicillin (100 U/ml) and streptomycin (100 μg/ml) (Life Technologies) as described previously [9]. The medium was replaced weekly and cells were passaged at least every 15 days. Cells derived from cultures of the same passage level were centrifuged, re-suspended in fresh complete medium to a concentration of 1 × 10^6^ cells/ml and seeded into new tubes (2 ml cell suspension per tube). Cultures were treated with a range of concentrations of analytical standard amitraz (Sigma-Aldrich, Milan, Italy), fipronil (Sigma-Aldrich) or permethrin (Sigma- Aldrich). Drugs were dissolved in dimethyl sulfoxide (DMSO) and then diluted in complete culture medium to final concentrations of 25, 50, 100 and 150 μM, maintaining the DMSO concentration at 0.5%. Control samples were treated with 0.5% DMSO only. Cultures were maintained for 10 days and medium was changed once on day 7. Experiments were carried out with four replicates per treatment protocol.

### Cell morphology, viability and cell density

After 10 days of incubation, live cell images were captured. Then, cells were resuspended and a small aliquot of cell suspension (0.3 ml) was harvested from each tube, labelled with 0.4 % w/v trypan blue and counted using a haemocytometer. The mean of four independent counts per tube was used to evaluate cell viability (live *versus* dead cell count) and density (total cell count), as previously described [23].

Cytocentrifuge smears were prepared with approximately 50 μl of cell suspension from control cultures and cultures treated with 150 μM acaricide, fixed in methanol and stained with a modified May Grünwald-Giemsa stain (Diff-Quik, Bio Optica, Milan, Italy).

MTT [3-(4,5-dimethylthiazol-2-yl)-2,5-diphenyltetrazolium bromide; Sigma-Aldrich] assays were also carried out at 10 days. Briefly, control and drug-treated cells were resuspended and 100 μl aliquots were transferred into a 96-well plate. Ten microlitres of MTT solution (dissolved at 5 mg/ml in complete L-15 medium) was added to each well and cells were incubated at 28 °C for 3 h. Plates were then centrifuged at 300× *g* for 5 min to sediment any cells in suspension. The medium was carefully removed and replaced with 100 μl of a lysis solution containing 10% w/v sodium dodecyl sulfate and 10 mM HCl and incubated overnight. Absorbance at 570 nm was measured with a Victor3 V plate reader (Perkin Elmer, Milan, Italy) and normalised against the absorbance at 650 nm. The mean of three independent experiments was calculated and each condition was compared to the DMSO-only control.

### Quantitative reverse-transcription PCR (qRT-PCR)

RNA was extracted from the remaining samples of re-suspended cells from each replicate culture using an RNeasy Mini Kit (Qiagen, Milan, Italy) following the manufacturer’s instructions. RNA was measured by spectrophotometric analysis for quality and content and then converted into complementary DNA (cDNA) using a QuantiTect Reverse Transcription Kit (Qiagen), according to the manufacturer’s instructions. The resultant cDNAs were used as templates for molecular analysis.

ABCB1, ABCB6, ABCB8 and ABCB10 genes and the endogenous control β-actin were detected by the qRT-PCR (CFX96 Touch™ Real-Time PCR Detection System, Bio-Rad, Milan, Italy), using the SsoAdvanced™ Universal SYBR® Green Supermix (Bio-Rad), and following the manufacturer’s instructions. As previously described [9], primers for four ABCT genes (ABCB1, ABCB6, ABCB8 and ABCB10) were designed based on conserved regions detected in the *Ixodes scapularis* and *R. sanguineus* transcriptomes (S. Epis, unpublished data). In synthesis, primer couples were tested in a traditional PCR protocol and reactions were run on a 2% agarose gel. The resulting amplicons were extracted, sequenced and deposited in the EMBL Nucleotide Sequence Database (ABCB1: LT222035; ABCB6: LT222036; ABCB8: LT222037; ABCB10: LT222038). Their expression in qPCR was normalised against the endogenous β-actin control (GeneBank: HQ682101).

The final concentration for each primer in all qRT-PCR reactions was 0.25 μM. The amplification protocol was characterised by a denaturation step at 98 °C for 2 min, followed by 50 repeated cycles (98 °C for 10 s, 57 °C for 15 s, 72 °C for 20 s). Fluorescence signals were collected in every cycle and the presence of nonspecific products was excluded through analysis of the melting curves. Results were presented as the mean of three independent experiments ± standard error of the mean, managed by CFX Manager software (Bio-Rad) and expressed as relative normalised expression (ΔΔCq).

### Data analysis

The means of three independent experiments with four replicates each were used to detect statistical significance in a one-way analysis of variance with Tukey’s *post-hoc* test in Past3 (v.3.14, http://folk.uio.no/ohammer/past/). A *P*-value < 0.05 was considered statistically significant.

## Results

### IRE/CTVM19 viability and metabolic activity

**Fig. 1:**
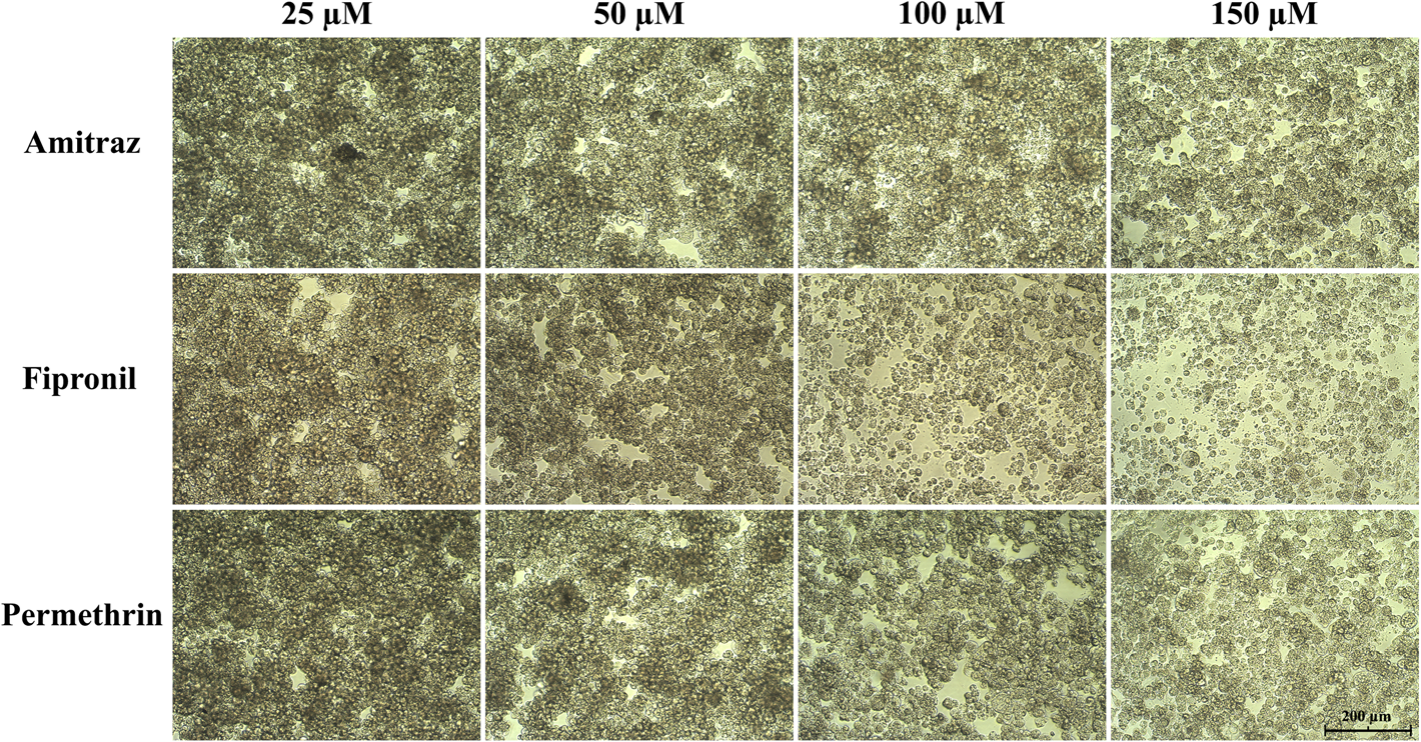
IRE/CTVM19 cells after 10 days of treatment with amitraz, fipronil or permethrin at concentrations of 25 μM, 50 μM, 100 μM and 150 μM. Higher drug concentrations induced changes in cell adherence and morphology, with cells appearing bigger and more vacuolated, and with a reduction in cell density. Pictures were captured using an IB2FL inverted microscope (Exacta Optech, Thame, UK) and OrmaEurotek camera (Orma Scientific, Milan, Italy) at 100× magnification. *Scale-bar*: 200 μm

**Fig. 2:**
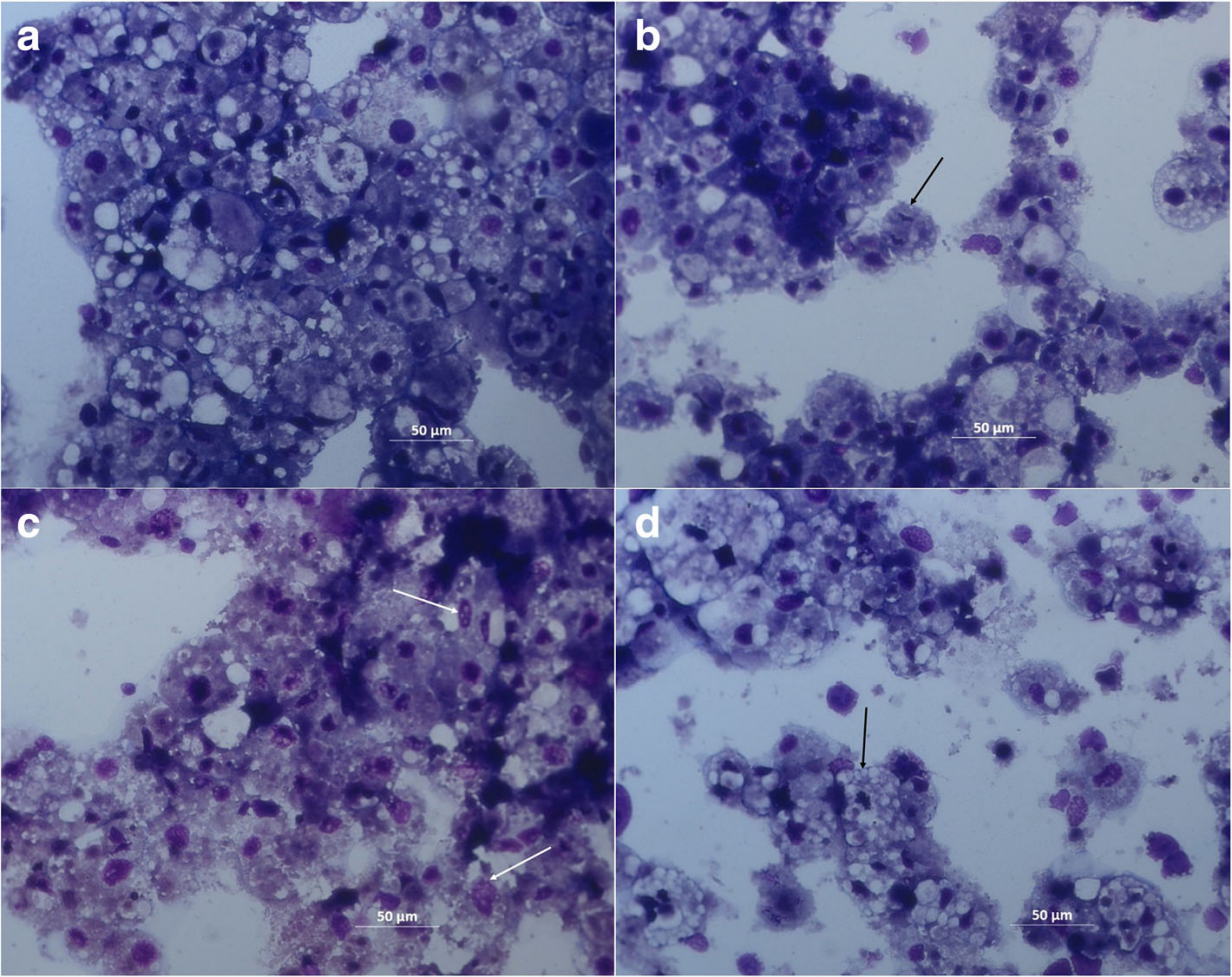
IRE/CTVM19 cells after treatment with 0.5% DMSO (**a**), 150 μM amitraz (**b**), 150 μM fipronil (**c**) and 150 μM permethrin (**d**), stained with modified May Grünwald-Giemsa (Diff-Quik) stain. Drug-treated cultures showed increased cellular debris, less well-defined cell borders and, in the case of fipronil, vacuolated nuclei (white arrows). Cells in mitosis are indicated by black arrows. Pictures were captured with brightfield illumination and a 25× objective using an Axio Imager M2 microscope with Axiocam MRc camera and Zen software (Zeiss, Milan, Italy). *Scale-bars*: 50 μm

All acaricides, when used at high concentrations (100–150 μM), resulted in decreased cell adherence and density, and increase in cell size and vacuolation (Fig. 1), while there was no difference in morphology of live cells between the lowest drug concentration and the untreated and the DMSO-treated control for all drugs (data not shown). Examination of stained cells (Fig. 2) revealed few obvious differences between control cultures treated with DMSO alone (Fig. 2a) and cultures treated with 150 μM amitraz (Fig. 2b) or permethrin (Fig. 2d), except that there were increased amounts of cell debris in the treated cultures and the margins of some treated cells were poorly defined. Cells in mitosis were seen in amitraz- and permethrin-treated cultures (Fig. 2b, d) as well as in controls (data not shown). In contrast, compared to cells treated with DMSO alone (Fig. 2a), cells treated with 150 μM fipronil showed markedly increased amounts of cell debris, no dividing cells, all intact cells had poorly-defined margins and lacked blue-stained cytoplasm, and many of the cell nuclei were vacuolated (Fig. 2c). Cell viability and metabolic activity of untreated and DMSO-treated control cells were not significantly different (data not shown).

**Fig. 3:**
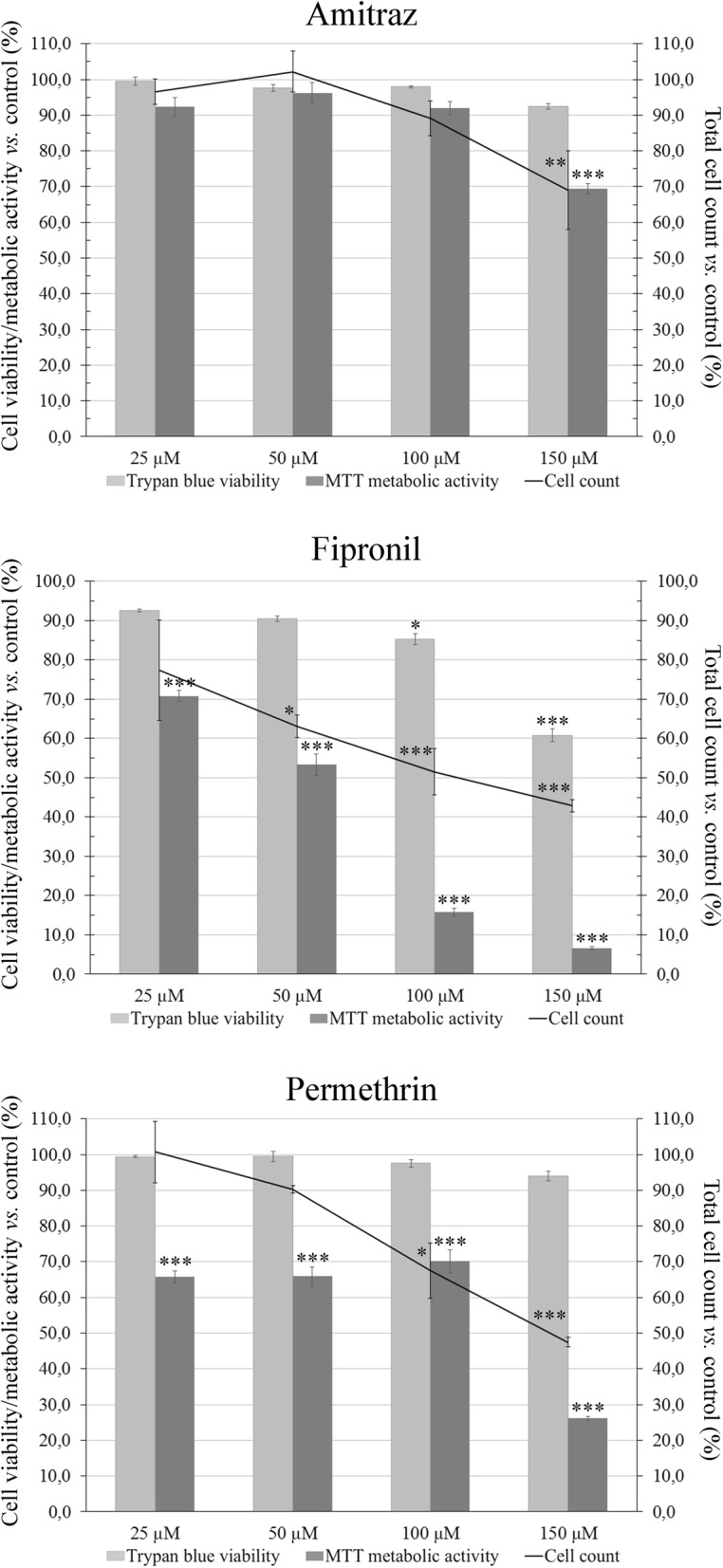
IRE/CTVM19 cell viability determined by trypan blue exclusion assay (light grey bars), metabolic activity determined by MTT assay (dark grey bars), and total cell count (black line) following 10 days of treatment with amitraz, fipronil or permethrin at concentrations of 25, 50, 100 and 150 μM. Data were reported as the mean of three independent experiments with four replicates each and expressed as percentage of the control (0.5% DMSO). *P < 0.05, **P < 0.01, ***P < 0.001

Trypan blue exclusion assays (Fig. 3) confirmed the reduction in total cell count (density) with increasing drug concentration observed by light microscopic examination of all treated cultures. There was no significant difference in viability of cells treated with amitraz at any concentration, when compared to cells treated with DMSO alone (*F*_(5, 66)_ = 1.987, *P* = 0.092). However, metabolic activity measured by MTT assay decreased at higher amitraz concentrations (*F*_(5, 66)_ = 34.19, *P* < 0.0001) and was significantly lower at 150 μM (*P* = 0.00012) (Fig. 3). Similarly, cells treated with permethrin did not show any decrease in viability at any concentration (*F*_(5, 66)_ = 2.123, *P* = 0.073); however, all concentrations of permethrin significantly decreased metabolic activity (*F*_(5, 66)_ = 234.6, *P* < 0.0001), which fell to 26.1% of the control level at 150 μM (*P* = 0.00012) (Fig. 3). Fipronil treatment induced a significant decrease in cell viability (*F*_(5, 66)_ = 159.4, *P* < 0.0001) at 100 μM (down to 88.7% of the control level, *P* = 0.00013) and 150 μM (down to 63.2% of the control level, *P* = 0.00013) and in metabolic activity at all concentrations (*F*_(5, 66)_ = 549.4, *P* < 0.0001, reduced by up 93% of the control level at the highest concentration, *P* = 0.00012) (Fig. 3). Metabolic activity determined by MTT assay (Fig. 3) did not always correspond to results from trypan blue exclusion assay, suggesting that a proportion of cells in cultures treated with permethrin and fipronil, and the highest concentration of amitraz, were alive but exhibiting a low level of metabolism.

### Induction of ABCB genes

**Fig. 4:**
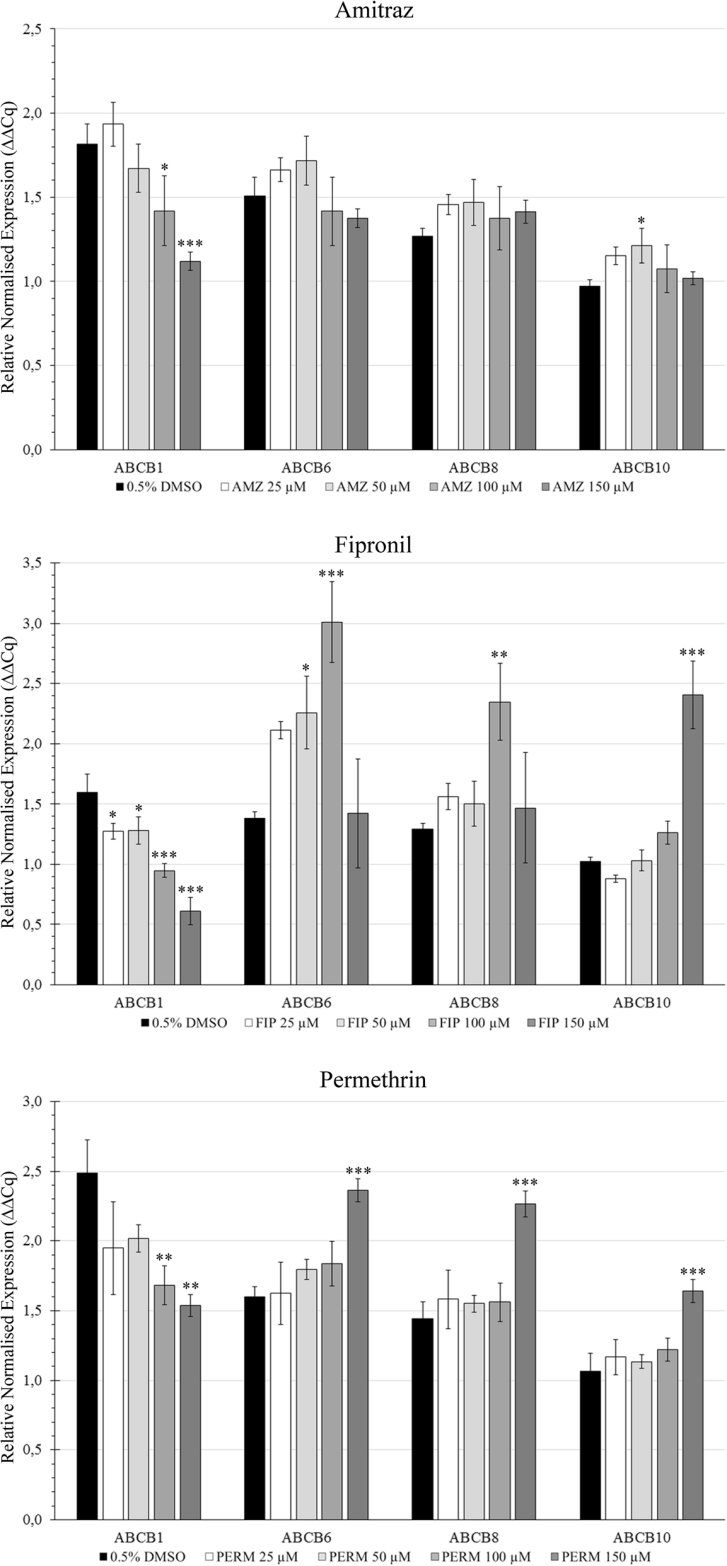
Expression of ABCB1, ABCB6, ABCB8 and ABCB10 genes in IRE/CTVM19 cells treated with amitraz, fipronil or permethrin at different concentrations. Data were reported as the mean of three independent experiments with four replicates each and expressed as relative normalised expression (ΔΔCq) *vs* time zero. *P < 0.05, **P < 0.01, ***P < 0.001

qRT-PCR revealed different patterns of expression of ABCB1, ABCB6, ABCB8 and ABCB10 genes in IRE/CTVM19 cells treated with amitraz, fipronil or permethrin at different concentrations (Fig. 4). The ABCB1 gene showed dose-dependent downregulation, which was highly significant at 150 μM with all three drugs.

Permethrin treatment induced significant upregulation of ABCB6 (*F*_(4, 10)_ = 15.52, *P* = 0.00027), ABCB8 (*F*_(4, 10)_ = 18.3, *P* = 0.00013) and ABCB10 (*F*_(4, 10)_ = 16.06, *P* = 0.00024) at 150 μM. Amitraz treatment showed slight modulation of all genes under investigation, with statistically significant upregulation of ABCB10 at 50 μM (*F*_(4, 10)_ = 3.978, *P* = 0.036). Fipronil treatment showed upregulated ABCB6 expression in a direct dose-dependent manner up to 100 μM (*F*_(4, 10)_ = 14.15, *P* = 0.0004) but had no effect at 150 μM (*P* = 0.2389); ABCB8 was upregulated at 100 μM (*F*_(4, 10)_ = 7.01, *P* = 0.005119) and ABCB10 was strongly upregulated at 150 μM (*F*_(4, 10)_ = 59.45, *P* = 0.00018).

## Discussion

An *in vitro* model for testing novel acaricides and examining the cellular mechanisms that they are able to activate, in order to evaluate the modulation of genes involved in drug resistance, would be beneficial in research on tick control. Previous studies have evaluated the effect in tick-derived cell lines of ivermectin on cell viability and cell detoxification [9, 10], and of coumaphos on various physiological parameters associated with acaricide resistance [24]. There are, however, no similar studies available for other acaricides in current use.

In the present study, the effect of treatment of *I. ricinus* cells with three commonly-used acaricides, amitraz, fipronil and permethrin, was evaluated. Cell morphology was assessed to determine if any of the drugs had an evident cytopathic effect at the doses used. The assessment of cell morphology in this *I. ricinus* embryo-derived cell line was challenging, as normal tick cell cultures comprise two or more cell types that can be present in varying proportions both at different times, within a single culture and at different passage levels [22]. In the present study, control cells showed a heterogeneous cell population with intact nuclei and cytoplasm with a variable degree of vacuolisation (Figs. 1 and 2a). Cells treated with the highest dose of the three acaricides showed signs of damage (increased extracellular debris, less well-defined cell borders, Fig. 2b-d) indicative of cell death, concurrent with a decrease in total cell count. These effects were most pronounced in the fipronil-treated cells, some of which also displayed vacuolation. There is no information about the acaricide resistance status of the female ticks from whose progeny this cell line is derived, nor about the tissue origins of the cell phenotypes present in the IRE/CTVM19 line. Furthermore, it is not known whether these include neuronal cells that might be expected to be adversely affected by acaricides targeting the tick nervous system. It is clear, however, that the cells treated with all three acaricides suffered considerable morbidity and/or mortality. This suggests that tick cells in general may be more susceptible to the effects of acaricides when isolated *in vitro*, or that factors present in whole live ticks may protect certain organs or tissues or prevent their exposure to acaricides administered topically or *via* the blood meal.

Differences in viability measured by trypan blue exclusion of surviving treated cells as compared to controls were negligible, with the exception of high doses of fipronil. On the contrary, metabolic activity measured by MTT assay gave markedly different results for fipronil and permethrin, with significant, dose-dependent reduction at all concentrations, suggesting that a proportion of the cells in these cultures were alive but with greatly reduced metabolism levels. Despite a decrease in total cell numbers, amitraz had little effect on metabolic activity of surviving cells except at the highest dose, suggesting that the mechanism of its effect on the tick cells may be different from that of the other two drugs.

It has been reported that MTT assays, which are based on MTT tetrazolium salt reduction to formazan through the activity of mitochondrial dehydrogenases, are more sensitive for determining cell metabolic activity (equivalent to viability) than dye exclusion which is based on permeability of dead cell membranes [25]. Trypan blue staining, therefore, cannot be used to distinguish between healthy tick cells and cells that are alive but losing cell functions.

Similar results of acaricide-induced cytotoxicity have been reported in cell lines derived from the insect *Spodoptera frugiperda* treated with permethrin [26] and in a *Drosophila melanogaster* embryo-derived cell line treated with fipronil [27]. It has been suggested that these *in vitro* toxic effects are due to oxidative stress and the subsequent activation of apoptosis [27–29].

The effect of acaricide treatment on ABC transporter protein gene expression was variable, depending on the protein and the drug. Treatment of cells with all three acaricides (fipronil, permethrin and amitraz) was consistently associated with downregulation of ABCB1. In mammalian cells, this transporter protein is present within the cell membrane and is responsible for detoxification of the cytoplasm [30]. It could be argued therefore that downregulation would result in drug accumulation in the tested tick cell line. Indeed, as mentioned above, dose-dependent effects on cell viability were evident in cells treated with fipronil and permethrin. However, downregulation of ABCB1 gene expression was not associated with cytotoxicity in cells treated with amitraz, indicating that there are likely to be other mechanisms involved in susceptibility/resistance to this acaricide, independent of cell membrane detoxification.

In the present study using a cell line derived from *I. ricinus*, the negative effects on cell viability and proliferation caused by fipronil and permethrin were associated with upregulation of ABCB6, ABCB8 and ABCB10 gene expression. Amitraz, on the other hand, had little or no effect on ABCT gene expression.

However, increased ABCB10 gene expression has been linked to acaricide resistance in two cell lines isolated from a different tick species, *R. microplus*: BME26 (*in vitro*-induced resistance to ivermectin) and BME/CTVM6 (parent ticks resistant to organophosphates, organochlorines and amitraz) [10, 11]. Another recent study [31] reported upregulation of ABCB10 expression *in vitro* in tick midgut cells derived from amitraz-resistant *R. microplus* ticks, thus making this gene a promising marker for monitoring acaricide resistance in this tick species. In the absence of antibodies specifically raised against tick ABC transporters, the latter authors used a commercial antibody reactive with part of the human P Glycoprotein (ABCB1) transporter to detect ABC transporter protein expression in freshly-harvested, isolated *R. microplus* midgut cells by immunofluorescence [31]. If this antibody also reacts with *I. ricinus* ABC transporter proteins, it could be used in future to determine if the changes in ABCB gene transcription observed in the present study following acaricide treatment are accompanied by corresponding changes in protein expression in IRE/CTVM19 cells.

While ABCB1 is present within the cell membrane and is responsible for detoxification of the cytoplasm, the other ABC transporter proteins examined in the present study are expressed on inner organelles, such as mitochondria (ABCB6 and ABCB8) and haemosomes (ABCB10), where they carry out various metabolic functions [30, 31]. Mitochondria play a central role in apoptosis. Increased generation of reactive oxygen species triggers the release of mitochondrial cytochrome c into the cytosol, which is considered a critical event that occurs during apoptosis [32–34]. It is therefore possible that the upregulation of mitochondrial ABCTs is due to effects of these drugs on mitochondria, including expression of cytochrome P 450 enzyme isoforms which are involved in drug metabolism [28, 29] or generation of reactive species that compromise mitochondrial function.

## Conclusions

To our knowledge, this is the first study to describe the effects of the commonly-used acaricides amitraz, fipronil and permethrin on cell viability, cell proliferation and ABCT gene expression in an *I. ricinus* tick cell line with the aim of developing an *in vitro* model for the evaluation of acaricide susceptibility/resistance. Further study is needed to identify the mechanism(s) through which cells die following exposure to acaricides and the role of ABCTs in resistance to these drugs.

## Abbreviations

ABC: ATP-binding cassette
ABCB: ATP-binding cassette, subfamily B
ABCT: ATP-binding cassette transporter
ANOVA: analysis of variance
cDNA: complementary deoxyribonucleic acid
DMSO: dimethyl sulfoxide
EMBL: European Molecular Biology Laboratory
GABA: γ-aminobutyric acid
qRT-PCR: quantitative reverse-transcription polymerase chain reaction
RNA: ribonucleic acid
SEM: standard error of the mean
TBPs: tick-borne pathogens
